# Demonstration of a Melanoma-Specific CD44 Alternative Splicing Pattern That Remains Qualitatively Stable, but Shows Quantitative Changes during Tumour Progression

**DOI:** 10.1371/journal.pone.0053883

**Published:** 2013-01-14

**Authors:** Livia Raso-Barnett, Balazs Banky, Tamas Barbai, Peter Becsagh, Jozsef Timar, Erzsebet Raso

**Affiliations:** 1 Department of Tumour Progression, 2^nd^ Institute of Pathology, Semmelweis University, Budapest, Hungary; 2 Department of Cellular Pathology, Guy’s and St Thomas’ Hospital, London, United Kingdom; 3 Tumour Progression Research Group, Hungarian Academy of Sciences, Budapest, Hungary; University of Tennessee, United States of America

## Abstract

The role of CD44 in the progression of human melanoma has mostly been characterised by qualitative changes in expression of its individual variable exons. These exons however, may be expressed to form a number of molecules, the alternative splice variants of CD44, which may be structurally and functionally different. Using real-time PCR measurements with variable exon specific primers we have determined that all are expressed in human melanoma. To permit comparison between different tumours we identified a stable CD44 variable exon (CD44v) expression pattern, or CD44 ‘fingerprint’. This was found to remain unchanged in melanoma cell lines cultured in different matrix environments. To evaluate evolution of this fingerprint during tumour progression we established a *scid* mouse model, in which the pure expression pattern of metastatic primary tumours, circulating cells and metastases, non-metastatic primary tumours and lung colonies could be studied. Our analyses demonstrated, that although the melanoma CD44 fingerprint is qualitatively stable, quantitative changes are observed suggesting a possible role in tumour progression.

## Introduction

Elucidation of the role of CD44 and its alternative splice patterns in melanoma biology has been challenging. Beyond its standard (CD44S), constitutively expressed region it has ten variable exons (v1–v10), forming the variable region (CD44v) [1], which potentially allows for the expression of thousands of different isoforms of different structure and function. At present, the expression of 42 CD44 isoforms has been confirmed at mRNA level, 29 of these have been shown to encode protein. Additionally, post-translational glycation adds a further layer of diversity to the possible protein structure and functions. These include binding to different components of the extracellular matrix, cytokine-binding and participation in signal pathways of cell growth and migration. [2]–[7].

Many of the variable exons’ individual functions have been examined individually demonstrating significant functional changes in signaling pathways. CD44 is the principal cell surface receptor for hyaluronate. When v3 is expressed [8] hyaluronate binding is weaker. Permitting further chondroitin and heparan sulphate glycosylation, results in the presentation of heparin-binding growth factors such as HB-EGF, b-FGF and amphiregulin. [9] For activating c-met by its ligand, HGF/SF, v6 is needed and important for the intracellular signal pathway via MEK and Erk. [10] In Ras pathway-regulated CD44 alternative splicing, those isoforms containing v6 together with HGF form a positive feedback loop on Ras, causing the downregulation of non-v6 containing isoforms [11].

However, there is discrepancy around the data on the role of CD44 and its isoforms in tumour biology. Despite the fact that it has been known for a number of years now that healthy tissues have specific CD44 Alternative Splice Patterns (ASPs) [12], and a PCR based method has already been proposed to analyse the different CD44 splice variants [13], there has not been any follow up to this study. Conversely, most data on CD44 alternative splicing in neoplasia and tumour progression have focused on the associations of the expression of single CD44 variable exons. There are problematic discrepancies in the role of CD44v in tumour biology.

For example, in colorectal carcinoma some studies indicate that v2 has a predictive role in progression [14], v3 and v6 play a role in cancer free survival [15] and v6 again in distant metastases [16], [17]. Other studies have failed to demonstrate any such role [18], [19], [20].

Similar discrepancies emerge in the changing patterns of CD44v expression in human melanoma. Dome et al. demonstrated the up-regulation of v3 results in a higher visceral metastatic potential [21] while Pacifico et al. showed that CD44v3 expression is associated significantly with a better outcome [22]. Again v10 [23] and v6 [24] may be involved in metastasis formation, or not [25]. One study suggests that human melanomas do not express CD44v isoforms at all and only express the CD44S form at a higher level, and does not correlate with the prognosis of this tumour type [26]. These varied findings may reflect the focus on the expression of solitary exons, rather than the profile of all ASPs.

Other studies do analyse the co-expression of two or more variable exons [27], [28], although not as a part of the ASP. In an alternative splice pattern, many different isoforms are present. The functional importance of any single variable exon may be dependent on the full expression pattern. Detecting the presence of a single, or multiple variable exons across all of these isoforms does not provide any information as to where these variable exons are expressed, and crucially what other variable exons are present alongside. On the other hand, the presence of additional variable exons on a particular isoform may actually change or not permit the function of the variable exon in question, and thus without knowing the entire alternative splice pattern, this restricts what one can say about detecting the presence of a single variable exon in these studies.

For the same reason even the ‘co-expression’ of two exons proven by immunohistochemistry [5], [17] does not mean that they are on the same molecule as the presence of two or more different CD44 isoforms in the same cell at the same time is also possible. Although the expression level changes of one variable exon might still show a correlation with the progression in one tumour type, there is no such obvious example in the literature as there are lots of contradictions even during the examination of the same tumour type.

Some more recent studies have analyzed the role of CD44v isoforms rather than single exons in tumour progression [29], [30], but not as a part of a complex, finely regulated pattern.

A more holistic view of the alternative splice event is needed to examine the role of CD44 variants.

This would be a huge practical challenge from tumour to tumour. We have sought to establish a reliable and reproducible method to examine this pattern and its possible tumour and/or progression specificity, since co-expression of exons proven by immunohistochanistry does not determine whether they are on the same molecule (and two or more CD44 may be present in the same cell at the same time) We have used a PCR based method using five primer pairs to create a simple representation of this highly complex CD44 expression pattern.

## Materials and Methods

### Cell Lines and Culture Conditions

The A2058 melanoma cell line was provided by LA Liotta (NCI, Bethesda, MD). HT168 and HT168M1 lines are derivatives of A2058 [31]. HT199 [31] was developed in the 1st Department of Pathology and Experimental Cancer Research (Semmelweis University, Budapest, Hungary). WM983B [32] and WM35 [32] were gifts from M. Herlyn (Wistar Institute, Philadelphia, PA). The colorectal carcinoma cell lines were HT25 [33] (from M. Hendricks, Iowa), HT29 (ECACC 91072201), HCT116 (ICLC HTL95025) and HCR31 [34]. We also used MDA-MB-231 human breast adenocarcinoma (ECACC 92020424), PE/CA PJ15 (ECACC 96121230) and PE/CA PJ41 (ECACC 98020207) human oral squamous cell carcinomas, K562 human chronic myelogenous leukaemia (ECACC 89121407) and A431 human vulva squamous carcinoma (ECACC 85090402) cell lines. The melanocyte (C-12403), keratinocyte (C-12003) and fibroblast (C-12360) cells were derived from Promo Cell. The melanoma and colorectal cell lines were maintained in RPMI 1640 medium supplemented with 10% fetal bovine serum (Sigma, St. Louis, MD), 2 mM glutamine, 0.1 mM non-essential amino acids, 1 mM sodium pyruvate, and 50 mg/ml gentamicin sulfate (all from Gibco BRL, Life Technologies, Paisley, Scotland). The melanocytes were maintained in Melanocyte Growth Medium M2 (PromoCell), the keratinocytes in Keratinocyte Media 2 (PromoCell) and the fibroblasts in Fibroblast Media (PromoCell).

### RT-PCR Analysis of CD44 mRNA Expression

Total RNA was isolated from the frozen homogenized tumour samples and cell cultures from the in vivo experiments using TRI Reagent™ (Sigma®) according to the manufacturer instructions**.** Possible DNA contamination was eliminated using TURBO DNA-free™ kit (Ambion®). For reverse transcription 1 µl of 10 mM dNTP mix (Finnzymes, Espoo, Finland) and 1 µl of random primer-oligo dT were mixed for a final concentration of 2.5 µM and used with 2 µg of purified total RNA. After incubating at 70°C for 10 min, 1 µl of M-MLV reverse transcriptase (200 units/µl), 2 µl of 10x M-MLV RT Buffer (both from Sigma), 0.5 µl RNase Inhibitor (40 units/µl, Promega, Madison WI) and 6.5 µl DEPC treated water was added for 20 µl final volume and incubated at 37°C for 50 min and then at 85°C for 10 min. The occurrence of reverse transcription was checked by polymerase chain reaction with β-actin primers (GTGGGGCGCCCCAGGCACCCA, CTCCTTAATGTCACGCACGATTTC) as a housekeeping gene. RNA of the same sample was used as negative control for detection of DNA contamination and DEPC treated water as non-template control.

### PCR Detection of CD44 Variable Exons

The PCR reaction mixture contained12,5 µl AmpliTaq Gold® 360 Master Mix, 2.5–2.5µl of the appropriate primer pair designed with Array Designer (Premier Biosoft International) (Figure S1). 2µl of the cDNA and 5.5 µl DEPC treated water for the final volume of 25 µl. The cycling conditions were: 97°C for 10 min once, then 95°C for 1 min, 55°C for 1 min, 72°C for 2 min for 35 cycles, 72°C for 10 min. The primer pairs were the following: S5’- variable exons3’, variable exons5’-S3’,

PCR products were separated using Experion™ Automated DNA 1K Kit1µl (Bio-Rad®) Electrophoresis System.

### PCR Detection and Sequencing of CD44 Fingerprint

The PCR conditions were as described above. The primer pairs were the following: S5’- S3’, S5’- v33’, v35’- S3’, S5’- v63’, v35’- v63’ using the exon specific primers (Figures S1, S2 and S3). PCR products were separated on a 3% agarose gel and detected with Gel Doc 2000 (Bio-Rad®) after ethidium bromide staining.

PCR products were re-isolated from the agarose gel (High Pure PCR Product Purification Kit, Roche, Mannheim) in the case of all bands. The DNA sequences were determined by BigDye® Terminator v1.1 Cycle Sequencing Kit (Applied Biosystems™ – by Life Technologies™).

### Cloning the PCR Products from the Fingerprint

The PCR products of the fingerprint were re-isolated from the 2% agarose gel (as above) inserted into vectors in the pGEM®-T Vector Systems (Promega ®) according to the manufacturer’s instructions. The plasmid DNA was re-isolated using GeneJET ^(^™^)^ Plasmid Miniprep Kit (Thermo Scientific) according to the manufacturer’s instructions and the products were sequenced with BigDye® Terminator v1.1 Cycle Sequencing Kit (Applied Biosystems™ – by Life Technologies™).

### Next-generation Sequencing

During a library preparation the ligated amplicon generation was used. The PCR products from HT199, A2058 and WM983B melanoma fingerprints were amplified, purified and their ends were polished and ligated with Roche 454 multiplex identifier (MID) adaptors, to generate universal primer binding sites for emulsion PCR in every sample reactions. The amplicons were than separated with magnetic Ampure beads (Agencourt) to eliminate the non-binded adaptors. Emulsification of the ligated emPCR was done according to the manufacturer’s protocol (Roche 454). The library was than sequenced with clonal pyrosequencing technic (454 GS Junior - Roche) with 200 cycles, in 400 bases read length mode. After sequencing, image processing and signal processing (amplicon pipeline) the amplicon variant analyzer software (Roche Diagnostics) was used to demultiplex the samples after MIDs, to trim the primers and to align the reads to the reference cDNA sequence. In the case of splice variants the difference between variants and the complete reference cDNA sequence can be too high. Therefore the reads were aligned to every exon separately to identify the real exon combination. The alignment was successful if there was higher identity than 90% in more than 20 bases length in every exons. The exon combinations which have more than 50 reads were reported.

### Culturing on Different Matrices

Fibronectin, laminin, collagen IV Matrigel, hyaluronate (each 50 µg/ml) and 0,9% NaCl solution (as control) were administered into different wells of a 6-well plate. After 3 hours of incubation on RT, supernatants were removed. 1–1 ml of 5×10^4^ cell/ml suspensions of HT168M1 was administered on the prepared matrix-films. After 72 hours of incubation, we removed supernatants, washed cell-films with EDTA, up-digested cell-films with tripsin-EDTA, collected up-grown cell suspensions and extracted total-RNA of cell masses with TRI-Reagent method.

### Metastasis Models Using *scid* Mice

This study was carried out in strict accordance with the recommendations and was approved by the Semmelweis University Regional and Institutional Committee of Science and Research Ethics (TUKEB permit number: 83/2009). All surgery was performed under Nembutal anaesthesia, and all efforts were made to minimize suffering.

Cultured HT199 and HT168M1 human tumour cells were injected subcutaneously (5x10^5^/50µl volume) at the same lower back localisation into 10 newborn and 10 adult *scid* mice as well as intravenously into 5 adult *scid* mice for both cell line. On the 30th day, the animals were sacrificed by bleeding under anaesthesia. Primary *in vitro* cell cultures were formed from the primary tumour, circulating tumour cells and the lung metastases of the same animal implanted as a newborn. Also, the primary tumour, circulating tumour cells and the i.v. transplanted lung colonies from the adult animals were used to create cell cultures the same way (Figure S4).

For comparative measurements the different tumours, i.e. primary tumour, circulating tumour cells, lung metastasis, always derived from the same animal to allow standardisation of the host.

### Quantitative PCR Analysis

For quantitative measurement of the expressed CD44 variable exons q-PCR reactions were used (iQ SYBR® Green Supermix,Bio-Rad), by cycling conditions 3 min at 95°C, then 40 cycles at 95°C 30 sec, 55°C 30 sec, 72°C 1 min. Starting quantities were defined on the basis of standard fivefold dilution series (1x-625X) carried out with control cDNA of A431 human squamous cell carcinoma) and by normalizing the starting quantities to the housekeeping β-actin starting quantities from the same cDNS sample. Three parallel measurements were carried out on each sample in every case.

## Results

### CD44 Variable Exons and Possible Isoforms at mRNA Level

**Figure 1:**
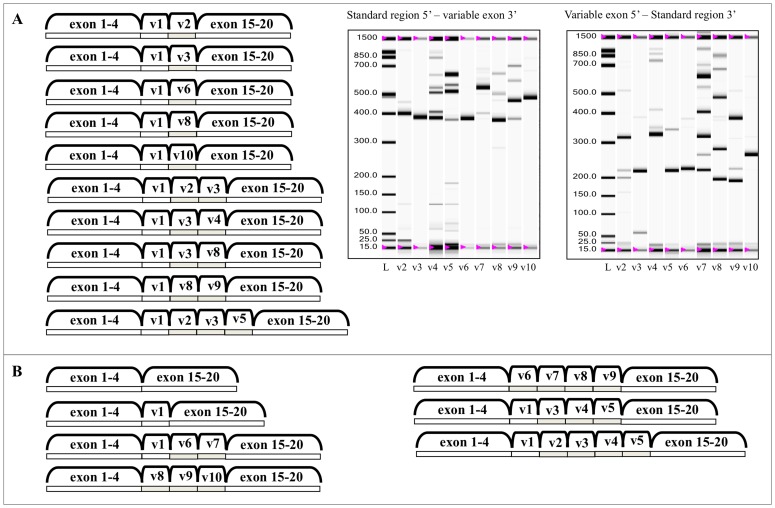
CD44 isoforms validated by next generation sequencing. **A.** CD44 isoforms from the qualitative picture of pairing the variable exon specific primers with the standard region specific ones both 5′ and 3′ directions in HT168 human melanoma cell line. These isoforms were validated by next generation sequencing. **B.** Further validated isoforms from next generation sequencing with the primer pairs of the fingerprint.

We visualized the expression of CD44 variable exons in HT168 human melanoma by performing PCR reactions pairing the sense (5′) primers of variable exons with the common antisense (3′) primer localized on exon 16 and variable exon’s antisense (3′) primers with the common sense (5′) on the standard exon 4. Our results showed, that all the variable exons, which are considered variable in databases (v2-v10) were present. Also, this method with the overlapping sequences allowed us to construct some of the isoforms (Fig. 1 and Fig. S5), although, this still seems rather inaccurate as some of the exons seemed to have been of slightly different size.

This size difference can possibly be explained by the fact that by next generation sequencing on the same tumour, we identified a daunting number of small deletions across the CD44 isoforms (data not shown).

**Figure 2:**
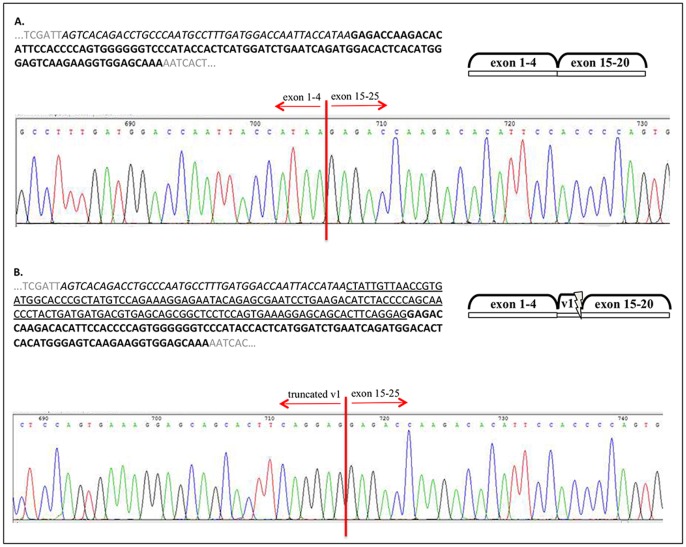
Cloned PCR products from the 5′ (exon 4, italic) and 3′ (exon 16, bold) primer (squared) combination of CD44 in A2058 human melanoma cell line. Direct sequencing shows a CD44 isoform with no v1 or any other variable exons (A) as well as one with truncated v1 (underlined).

We made further attempts and cloned our PCR products from A2058 and HT168 M1 human melanoma cell lines, which resulted in certain isoforms being more dominant and inserting at a higher rate, but yet again, the full set of the expected/calculated isoforms could not be identified. However, direct sequencing of some of the cloned sequences confirmed that v1, is in fact missing in some of the isoforms, which tied in nicely, with our above mentioned PCR-based results (Fig. 2A). Furthermore, some isoforms contained a truncated version of v1 (Fig. 2B).

### The CD44 Melanoma Fingerprint

In light of the complexity of CD44 isoform expression simple method to represent this pattern was developed which included v3 and v6– the exons considered to be of importance for melanoma progression.

For this purpose, we designed a five primer pair containing PCR-reaction series using a certain combination of the exon specific primers, which covered the whole variable region with five overlapping sequences. The first primer pair was located on the two standard regions allowing detection of all variable exons (S5’–S3’, primer pair 1). The second was on exon 4 of the standard region and v3, which makes possible to detect v2-v3 co-expression (S5’-v3’, primer pair 2). The third straddles the v3-v10 region by binding to v3 and exon 16 of the standard region (v35’-S3’, primer pair 3). The fourth pair was designed to exon 4 and v6 detecting the co-expression of v2–v5 with v6 (S5’-v63’, primer pair 4). The fifth one was detecting v4–v5 expression of v3–v6 co-expressing isoforms by binding to v3 and v6 (v35’-v63’, primer pair 5). The five PCR products of the same sample were run always in this same order in every case, so the pattern of the bands were comparable across all of our samples in the different experimental models. This bar code-like pattern is what we define as the ‘fingerprint’, a simplified representation of the CD44 ASP.

**Figure 3:**
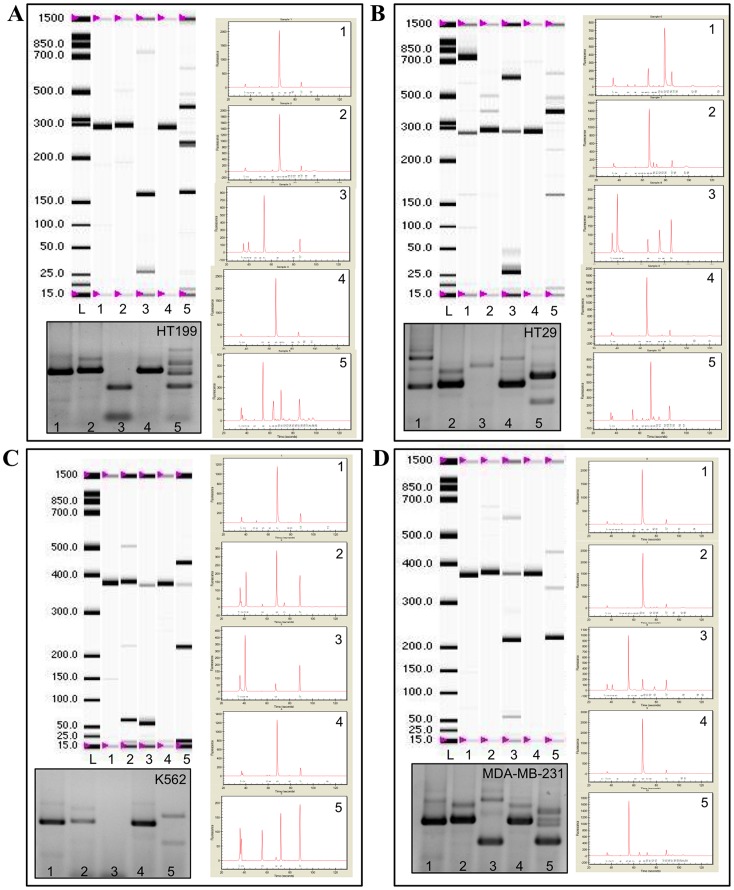
The CD44 alternative splice pattern of different human tumour cell lines demonstrated by virtual gels and electropherograms generated by Experion DNA Capillary Electrophoresis System and corresponding agarose gel picture. **A.** HT199 human melanoma cell line **B.** HT29 human colorectal adenocarcinomacell line **C.** K562 human erythromyeloblastoid leukemia cell line **D.** MDA-MB-231 human breast carcinoma cell line.

**Figure 4:**
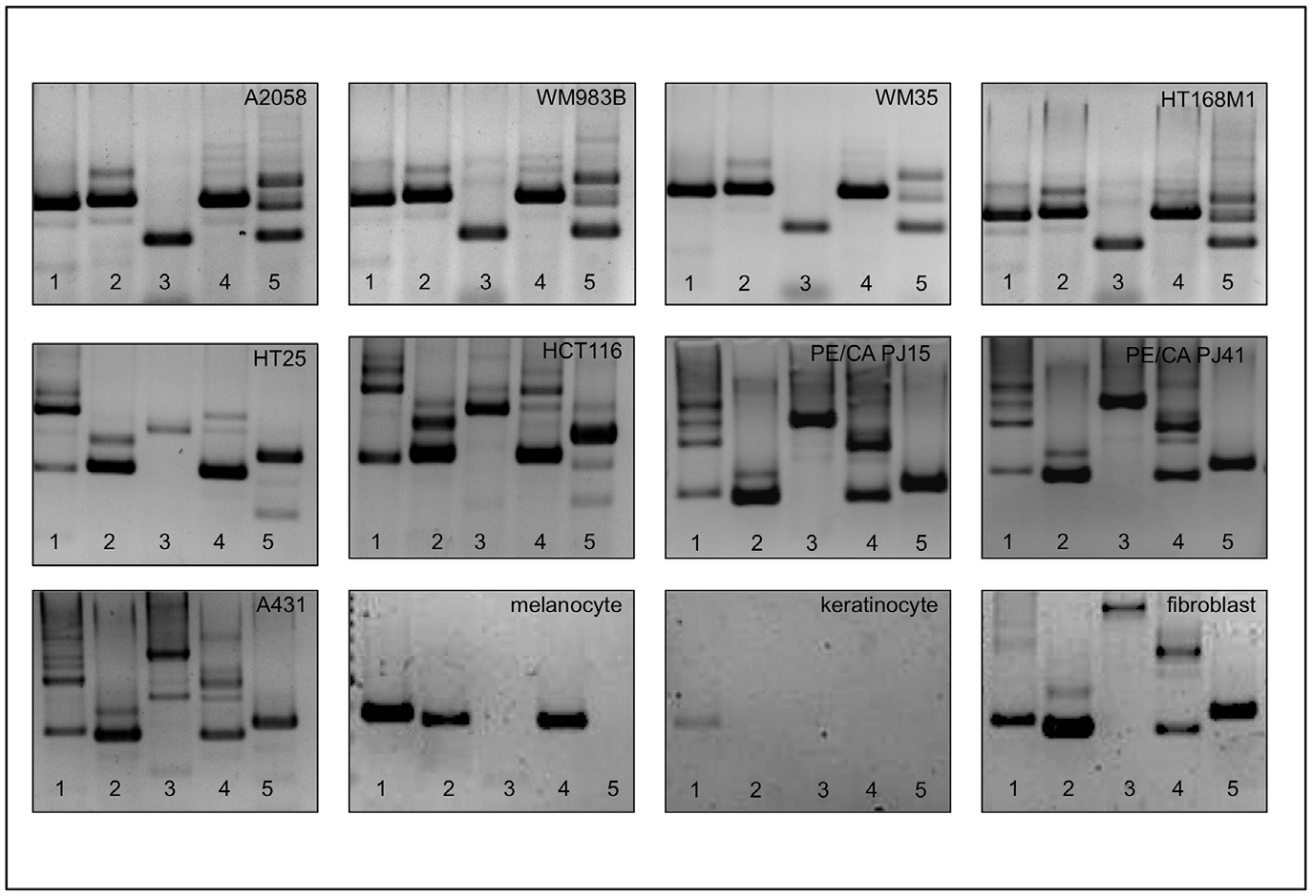
The CD44 alternative splice pattern of different human tumours is different, but preserved throughout samples from the same the tumour type as it is demonstrated by the agarose gel electropherograms of human melanoma (A 2058, WM983B, WM35 and HT168M), colorectal adenocarcinoma (HT25 and HCT116), oral squamous cell carcinoma (PE/CA PJ15 and PE/CA PJ41) and vulval squamous cell carcinoma (A431) cell lines. The melnanoma CD44 fingerprint also differs from that of non neoplastic melanocyte, keratinocyte and fibroblast cell lines as constituents of the microenvironment.

With this method we examined the CD44 ASP of human melanoma cell lines (HT168M1, WM35, WM983B, A2058 and HT199) in culture to establish whether there is a pattern that is conserved across these genetically different tumours. As it is shown in Fig. 3A and 4, we found a consistent pattern throughout, which we refer to as the melanoma fingerprint. Some of the isoforms, which were predicted from the melanoma fingerprint, based on the size of the bands, were confirmed by next generation sequencing. Although the reading frame of 454 GS Junior is significantly wider than that of similar techniques, its higher limit is still 400–700 bp. Therefore, even though next generation sequencing is rather promising, we are still relying on estimations in the case of larger products. 10 isoforms were confirmed (Fig. 1A) and a further 26 predicted (Figure S5) as part of the melanoma CD44 fingerprint.

We then compared this pattern to that of other human tumour cell lines grown in culture. These included cell lines derived from human colorectal adenocarcinoma (HT29, HT25, HCT116), human oral squamous cell carcinoma (PE/CA PJ15 and PE/CA PJ41), vulval squamous cell carcinoma (A431) and K562 human erythromyeloblastoid leukemia cell lines.Comparison was also made with primary cultured human melanocytes, skin keratinocytes and skin fibroblasts (Fig. 4). In each case the fingerprint differed unambiguously from the melanoma fingerprint, raising the possibility of a melanoma specific isoform expression pattern.

### Modeling the Effects of the Microenvironment *in vitro*

**Figure 5:**
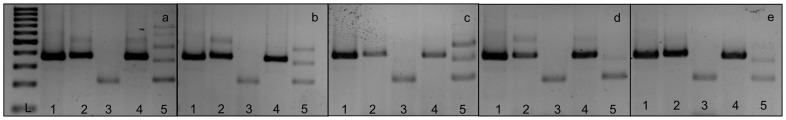
CD44 ‘fingerprint’ of HT168M1 human melanoma cell line growing on different matrices namely plastic (a), fibronectin (b), laminin (c), collagen (d) and matrigel (e). L stands for molecular weight marker.

To decide whether the *in vitro* melanoma CD44 fingerprint is maintained *in vivo* despite the influence of the microenvironment, we compared the CD44 splicing pattern of several, genetically different human melanoma cell lines (A2058, HT199, WM35, WM983A, M35) growing on plastic or different matrices. We also investigated HT168, a cell line cultured from the *in vivo* xenograft variant of A2058; HT168M1, a cell line which is the *in vivo* selected metastatic version of HT168; WM983B, cultured from a lymph node metastasis from the patient whose primary tumour gave rise to WM983A. Since CD44, as a cell surface glycoprotein, plays an important role in cell-matrix interaction, it was important to examine whether different matrix components change the alternative splicing pattern, or whether the ASP is stable and possibly inherent to melanoma-specific behavior. Therefore as a first step we determined the CD44 fingerprint of HT168M1 human melanoma cell line growing *in vitro* on different matrices, namely fibronectin, laminin, collagen and matrigel. As shown in Fig. 5 after 48 hours incubation time the CD44 fingerprint was found to be unchanged in the case of every matrix type (Fig. 5). This fingerprint was found to be consistent through all examined cell lines growing on different matrices (only HT168M1 shown). It is interesting, that the fingerprint is retained in the cell lines derived from the primary tumours and their metastases alike (HT168 versus HT168M1 and WM983A versus WM983B).

### The CD44 Melanoma Fingerprint *in vivo* in Our Animal Model

As the *in vivo* microenvironment is far more complex than the influences of the extracellular matrix, we used an animal model to evaluate the CD44 melanoma fingerprint *in vivo*. This model has been developed by our group, following the observation that semi-orthotopically (subcutaneously) implanted human melanomas always formed metastases in newborn *scid* mice (permissive host), yet never did so in adult ones (nonpermissive host). This model made it possible to examine the melanoma ‘fingerprint’ during the metastatic processes.

In vivo expression patterns were evaluated on two human melanoma cell lines HT199 and HT168M1.

We performed our PCR reaction series on theprimary subcutaneous tumour, circulating tumour cells obtained from blood and lung metastases from transplanted newborn *scid* mice, as well as the primary subcutaneous tumours from transplanted adult mice. In addition lung tumours were generated in adult animals by intravenous injection (Fig. S4).

**Figure 6:**
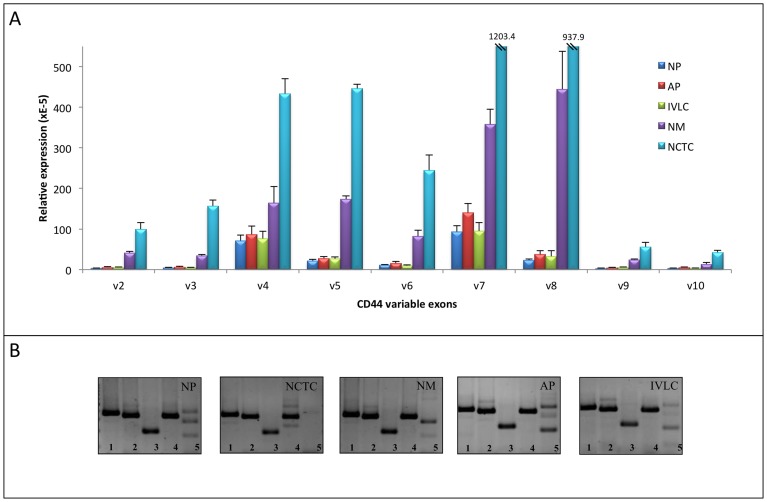
Relative quantitative expression of CD44 variable exons in cell cultures from metastatic (newborn) and non-metastatic human xenograft model (Real-Time PCR measurement) of HT199, a human melanoma cell line of originally low variable exon expression level. **A.** The relative expression level of all variable exons is raised in circulating metastatic cells (NCTC) and metastatic cells (NM) compared to their levels in primary tumours [newborn primary (NP) and adult primary (AP)] and lung colony (IVLC) **B.** The qualitative fingerprint (bottom line) remains unchanged.

For HT199 we found that the CD44 fingerprint demonstrated *in vitro* was unchanged throughout the sampled sites (Fig. 6B). These findings do not explain published observations, that the expression of certain CD44 exons correlate with metastatic potential. Our results suggest that the CD44 ASP behind the ‘fingerprint’ is the same in all these cases, meaning that the same isoforms are present. The cited quantitative expression changes of single variable exons should therefore be explained differently.

We made a further quantitative PCR analysis with our variable exon specific primers on the same samples. We examined the quantitative changes of the individual variable exons (VE) during tumour progression in the *in vivo* animal models of two genetically different human melanoma cell lines (HT199, HT168M1). It has become clear after the first measurements, that the two cell lines have 3 orders of magnitude difference in their CD44 VE expression relative to beta-actin housekeeping gene, but despite this, their malignant potential was practically identical. CD44 in HT199, the cell line with a low base VE expression, behaved as a ’classical’ metastasis gene. The non-metastatic adult primary (AP), the metastatic newborn primary (NP) and the lung colony formed after intravenous injection into adult animals (IVLC), which is also a form of primary tumour, all expressed the VEs within the same order of magnitude (Fig. 6A). The circulating tumour cells (NCTC) and lung metastases (NM) from the animal implanted as a newborn showed 21 times and 9 times increased expression respectively.

**Figure 7:**
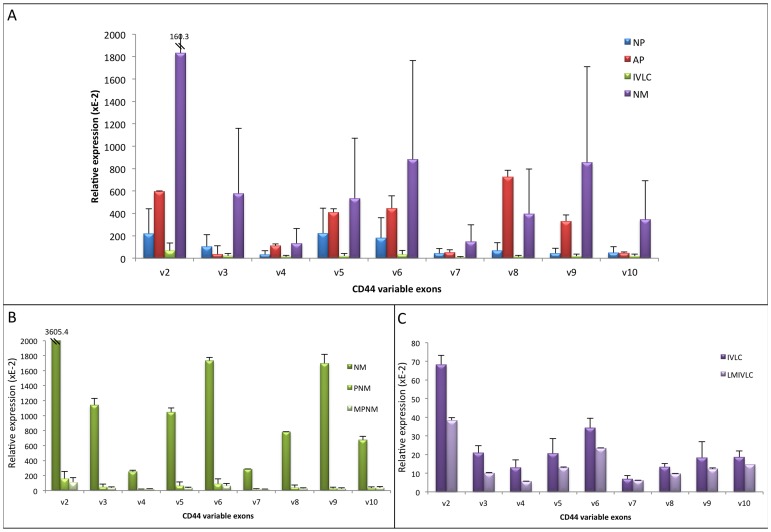
Relative quantitative expression of CD44 variable exons in cell cultures from metastatic (newborn) and non-metastatic [adult (AP)] human xenograft model (Real-Time PCR measurement) of HT168M1, a human melanoma cell line of originally high variable exon expression level. **A.** The relative expression level of all variable exons is raised in certain lung metastasis (NM), when remaines low or even decreases in other lung metastases resulting in large error bars. It should be noted that the expression level of the primaries from different localisations [newborn primary (NP), adult primary (AP) and intravenously implanted lung colony (IVLC)] are all comparable, although the slightly higher expression observed in the adult primary is an unexpected finding. **B.** We used the lung metastasis from the newborn animal with the highest CD44 variable exon expression level (NM = S1T2) for subcutaneous re-implantation into another newborn animal. The expression level in the primary tumour (PNM) and its metastases (MPNM) was 24 times lower on average. **C.** The liver metastases (LMIVLC) from the intravenously implanted lung colonies (IVLC) showed a decrease in expression, when compared to the lung colonies (IVLC).

In the case of HT168M1, which expresses the CD44 VEs in 3 times larger order of magnitude than HT199, the role of CD44 is more complex. In this case, we created cell cultures from different localisations of the primary tumour of individual animals. The individual lung metastases (newborn animals) and lung colonies (adult animals) of the individual animals were also cultured separately. The non-metastatic adult primary tumour (AP) showed a higher expression level of all CD44 VEs than the metastatis newborn primary tumour (Fig. 7A). The reason behind the rather large error bars seen on the measurements from the newborn lung metastases (NM) is that the individual lung metastases showed huge VE expression level differences. Cells from the cell line created from the lung metastasis showing the highest CD44 VE expression level (NM = S1T2, Figure 7B) were then re-implanted subcutaneously into newborn animals. Cell cultures were then created from three different localisations of the primary tumour (PNM) and three random lung metastases (MPNM) of the chosen animal (Fig. S4). They showed no difference in CD44 VE expression level compared to each other, however they showed on average 24 times lower expression than the cell culture (newborn lung metastasis, NM = S1T2) of origin. We also consistently detected lower CD44 VE expression in liver metastases (LMIVLC) from lung colonies (IVLC), which are practically secondary metastases (Fig. 7C).

## Discussion

Due to the possibility of a large number of different CD44 isoforms present at the same time in the examined sample, it was important to establish a method that would give a good representation of them.

Our first step was to determine which variable exons, other than the ones most studied in the literature, are expressed at mRNA level in human melanoma. We showed that all the variable exons are expressed in human melanomas and predicted a number of paralelly expressed CD44 isoforms. We also found that v1 was missing from some of the isoforms, although it is not considered as a variable exon, also that some of the isoforms contained a truncated v1 exon. This was confirmed by direct sequencing of our cloned molecules. However, next generation sequencing studies have surfaced a whole other level of ‘complications’ by identifying a number of deletions across the variable exons.

As a surrogate for representation of all of the expressed alternative splice variants of CD44, we developed a method to determine a ‘fingerprint’ of expression in human melanoma. This appeared to be stable in cell culture and mouse xenograft models and differed substantively from that found in colorectal adenocarcinoma, squamous cell carcinoma and primary cultures of human melanocytes, kerationcytes and fibroblasts. This technique bypasses the attempts to link the expression or co-expression of individual variable exons to metastasis formation, a strategy which loses crucial contextual information about the complex ASP underlying the CD44 protein set. This oversimplification may also account for some of the contradictory evidence on the associations of CD44 expression with the generation of a metastatic phenotype [35], [36].

Previous work in our laboratory has shown that under the effect of host derived selection factors, xenografts of tumour cells growing in new born *scid* mice differ in gene expression pattern than those growing in adult mice. It is not yet clear whether this pattern is related to formation of metastasis or reflects a summation of changes resulting from local effects of graft:host interaction. Adaptation to a new microenvironment is a crucial factor in the formation of metastasis: This may require events in changing patterns of gene expression prior to implantation or may reflect post hoc modification of expression in response to the metastatic niche. To be able to study the pattern of expression during tumour progression we have established an experimental mouse model in which the expression pattern of pure cultured cells from a primary implanted tumour, circulating cells in the peripheral blood stream and cells within established metastases in newborn *scid* mice from the same, individual animal could be studied. In addition expression patterns could be compared with those generated in adult *scid* mice either as primary tumours as lung colonies since spontaneous metastases are not formed in this animal population. We followed the CD44 VE expression changes during tumour progression of two human melanomas, that express CD44 VEs at different orders of magnitude, in this experimental animal model. We found that CD44 VE expression and metastasis formation showed inverse correlation, similarly to our recent findings in colorectal carcinomas [37]. The adult primary tumour, newborn primary tumour and lung colony of HT199, the human melanoma cell line with low base CD44 VE expression level, all expressed the variable exons in the same order of magnitude and within the error bar. However, as only a limited number of cells within the primary tumour are capable of forming metastasis, the above finding is not surprising. The dramatic increase in CD44 VE expression level seen in the metastatic tumours means, that the variants most likely play a role in metastasis formation. The extreme high level of CD44 VE expression in the circulating tumour cells of the newborn animals seems to indicate, that their role is mainly in getting into and/or surviving in the circulation. It seems, that for the new population of metastatic cells in the target organ, CD44 VE expression level is not as much or even not at all important.

The CD44 VE expression level in HT168M1, which has a high base CD44 VE expression, varies within the metastases, ranging from barely detectable to approaching the level detected in circulating tumour cells. When a population with extreme high CD44 VE level is then re-implanted, the expression level dramatically decreases in the metastases while the qualitative picture (fingerprint) remains unchanged.

From these results, we suggest that predicting the role of CD44 variable exon expression in tumour progression is more complex than previously anticipated. Our qualitative studies identified a melanoma-specific CD44 ASP, or fingerprint, which is different to the pattern of other examined tumour types. While this certainly raises the possibility to use this fingerprint to identify the unknown primary of metastases, the stability of this fingerprint during tumour progression also shows that no isoforms changes occur that could be correlated to metastatic phenotype or prognosis. However, our real-time PCR measurements suggest that quantitatively, melanoma is not a uniform group and CD44 variable exon expression levels can follow different patterns. Primary tumours with low overall CD44 VE expression level harbor metastatic clones with high expression level, which appears to be needed for entering the circulation and forming metastases. On the other hand, primary tumours with high base CD44 VE expression also contain metastatic clones, that either have sufficient CD44 expression or ‘utilize’ other molecules to facilitate metastasis formation. This, however, does not explain the extreme low CD44 VE expression levels detected in lung metastases, and further research in this area is needed. In any case, in both scenarios the CD44 VE expression level of the true metastatic clone is not immediately obvious from the overall expression of the primary tumour, which partially explains the contradicting results described in the literature.

We hypothesise that the metastatic clone is not the result of ratio changes or even an ’appearing/disappearing variable exon’, which might be one of the several factors to give metastatic property to that clone. Ultimately, these results show that even in one of the ‘most simple’ CD44 fingerprints, melanoma, we cannot talk about ‘CD44’ as a single molecule anymore.

## Supporting Information

Figure S1 **Sequence and localisation of the exon specific primers.**
(TIF)

Figure S2 **Localisation of the primer pairs used to create the fingerprint.**
(TIF)

Figure S3 **The melanoma fingerprint.** **A**. Fingerprint with the product sizes. **B.** Predicted isoforms based on the qualitative fingerprint. From the qualitative picture, the following isoforms can be identified in melanomas: CD44S which does not contain variable exons and appears as 372bp product in lane 1 of the fingerprints (a); CD44v3 containing only v3 exon appearing as 385 bp product on lane 2 and 220 bp product in lane 3 of the fingerprint (b); CD44v6 with also one variable exon (v6) as the 379 bp product of lane 4(c); CD44v2v3 with two expressed variable exons, v2 and v3 represented by the 514 bp product of lane 2 (d); CD44v3v6 is also two variable exon containing isoform which can be identified from the 627 bp product of lane 1, the 505 bp product of lane 4 and the 227 bp product of lane 5 (e) and CD44v3v4v5v6v9 as the biggest isoform with five expressed variable exons detected as the 670 bp product of lane 3, the 736 bp product of lane 4 and the 458 bp product of lane 5 (f). As the variable exons are very similar in size with sometimes only a few base pair difference other isoforms might be present as well and the presence of v7, v8, v9 and v10 is also possible. For instance the 204bp long v10 and the 207 bp long co-expressed v5 and v9 are very hard to distinguish as they would appear as ‘one’ band on lane three and only v5v9 co-expression can be proved by the appropriate sized products of lanes 4 and 5. This is further confirmed by cloning and next generation sequencing.
(TIF)

Figure S4 **Schematic structure of the** ***in vivo*** **human melanoma (HT199 and HT168M1) metastasis animal model.** The same melanoma cell suspension was implanted subcutaneously into adult and newborn *scid* mice as well as intravenously into adult *scid* mice. The primary adult [(subcutaneously (AP) and i.v. implanted (IVLC)] and newborn tumours (NP) were removed along with the liver (NM) and lung (NLM) metastases, that were only formed in newborn mice, on the 26^th^ post-implantation day. Cell cultures were created from all the above tumours and the circulating tumours cells (NCTC) of newborn mice. A cell culture created from a single HT168M1 lung metastasis of a newborn mouse was then re-injected subcutaneously into newborn *scid* mice and the primary tumour (PNM) and its lung metastasis (MPNM) were also removed and cultured on the 26^th^ post implantation day.
(TIF)

Figure S5 **Further Hypothesized CD44 isoforms.** **A.** Hypothesized CD44 isoforms from the qualitative picture of pairing the variable exon specific primers with the standard region specific ones both 5′ and 3′ directions in HT168 human melanoma cell line. **B.** Hypothesized isoforms using next generation sequencing with the primer pairs of the fingerprint
(TIF)
