# Supplementary figures and images for: Demonstration of a Melanoma-Specific CD44 Alternative Splicing Pattern That Remains Qualitatively Stable, but Shows Quantitative Changes during Tumour Progression
Source: PLoS One. 2013 Jan 14;8(1):e53883. doi: 10.1371/journal.pone.0053883 (PMC3544768; doi:10.1371/journal.pone.0053883)

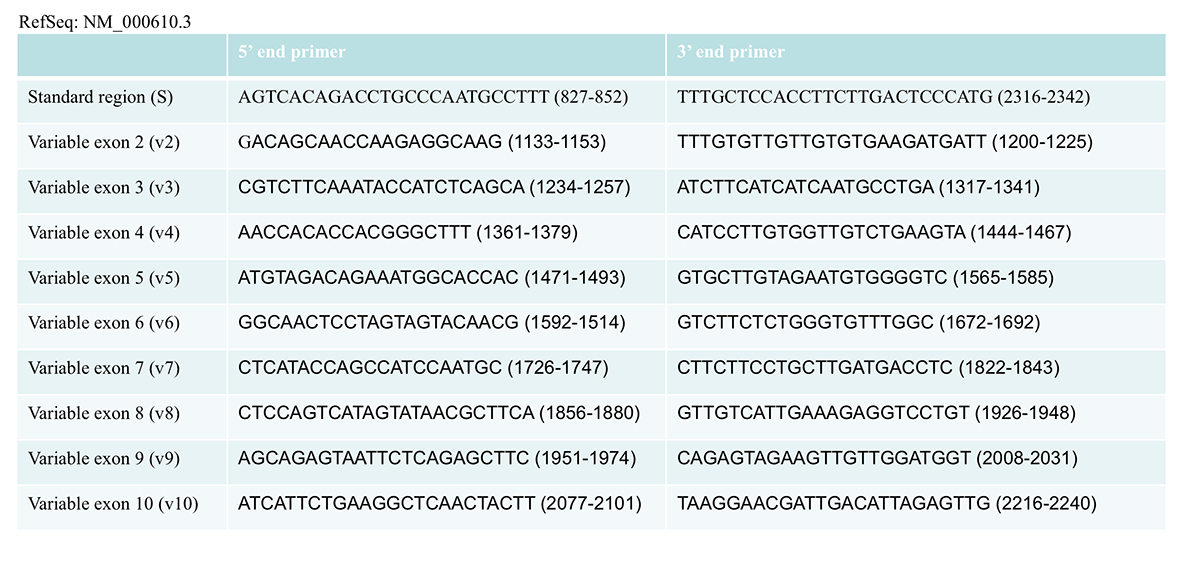

Supplement: Figure S1 — Sequence and localisation of the exon specific primers. (TIF) [file pone.0053883.s001.tif]

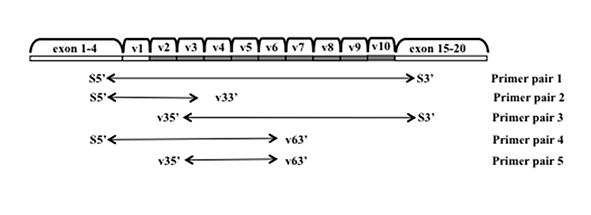

Supplement: Figure S2 — Localisation of the primer pairs used to create the fingerprint. (TIF) [file pone.0053883.s002.tif]

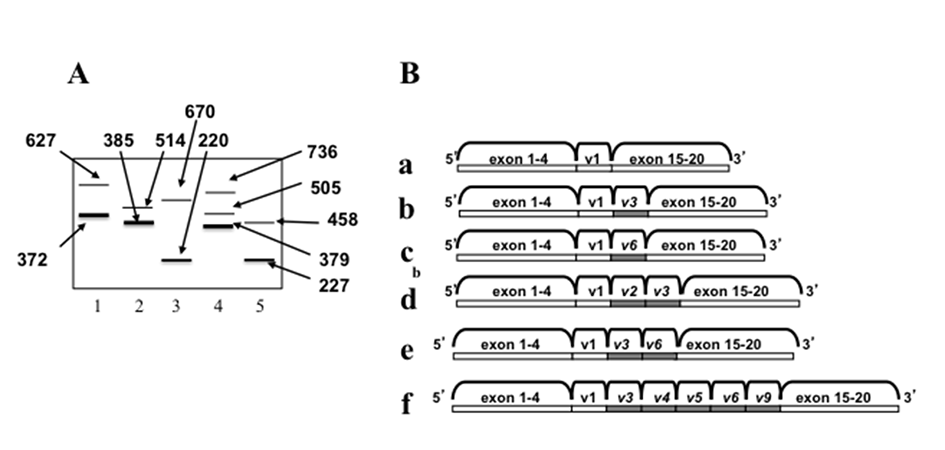

Supplement: Figure S3 — The melanoma fingerprint. A. Fingerprint with the product sizes. B. Predicted isoforms based on the qualitative fingerprint. From the qualitative picture, the following isoforms can be identified in melanomas: CD44S which does not contain variable exons and appears as 372bp product in lane 1 of the fingerprints (a); CD44v3 containing only v3 exon appearing as 385 bp product on lane 2 and 220 bp product in lane 3 of the fingerprint (b); CD44v6 with also one variable exon (v6) as the 379 bp product of lane 4(c); CD44v2v3 with two expressed variable exons, v2 and v3 represented by the 514 bp product of lane 2 (d); CD44v3v6 is also two variable exon containing isoform which can be identified from the 627 bp product of lane 1, the 505 bp product of lane 4 and the 227 bp product of lane 5 (e) and CD44v3v4v5v6v9 as the biggest isoform with five expressed variable exons detected as the 670 bp product of lane 3, the 736 bp product of lane 4 and the 458 bp product of lane 5 (f). As the variable exons are very similar in size with sometimes only a few base pair difference other isoforms might be present as well and the presence of v7, v8, v9 and v10 is also possible. For instance the 204bp long v10 and the 207 bp long co-expressed v5 and v9 are very hard to distinguish as they would appear as ‘one’ band on lane three and only v5v9 co-expression can be proved by the appropriate sized products of lanes 4 and 5. This is further confirmed by cloning and next generation sequencing. (TIF) [file pone.0053883.s003.tif]

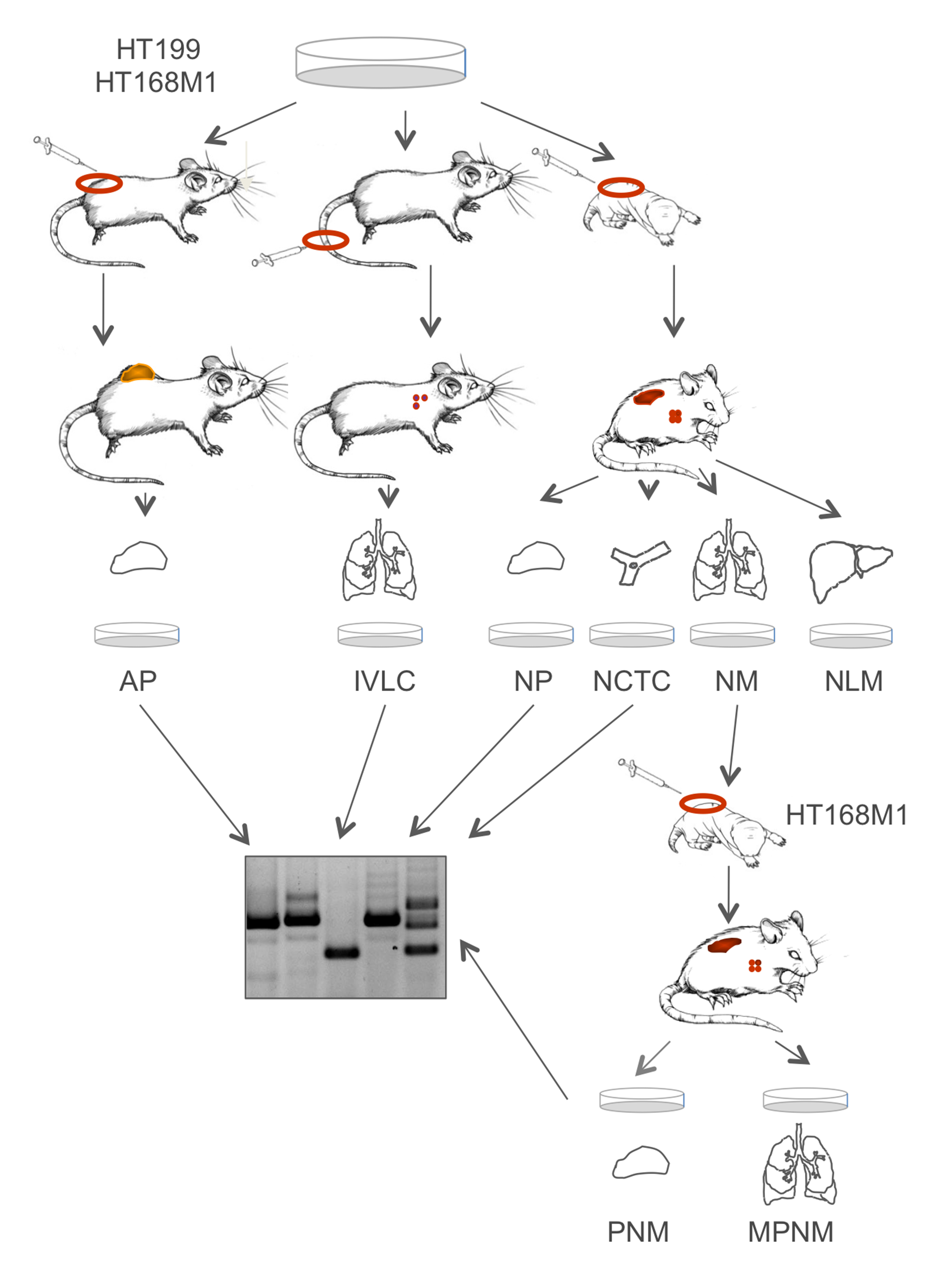

Supplement: Figure S4 — Schematic structure of the in vivo human melanoma (HT199 and HT168M1) metastasis animal model. The same melanoma cell suspension was implanted subcutaneously into adult and newborn scid mice as well as intravenously into adult scid mice. The primary adult [(subcutaneously (AP) and i.v. implanted (IVLC)] and newborn tumours (NP) were removed along with the liver (NM) and lung (NLM) metastases, that were only formed in newborn mice, on the 26th post-implantation day. Cell cultures were created from all the above tumours and the circulating tumours cells (NCTC) of newborn mice. A cell culture created from a single HT168M1 lung metastasis of a newborn mouse was then re-injected subcutaneously into newborn scid mice and the primary tumour (PNM) and its lung metastasis (MPNM) were also removed and cultured on the 26th post implantation day. (TIF) [file pone.0053883.s004.tif]
